# Endosymbiotic interactions in the fungal kingdom: a framework for progressive endosymbiosis

**DOI:** 10.3389/fmicb.2026.1864871

**Published:** 2026-06-15

**Authors:** Juan Jose Hernandez-Gonzalez, Elva Teresa Arechiga-Carvajal, Olga Miriam Rutiaga-Quiñones, Katia Jamileth Gonzalez-Lozano

**Affiliations:** 1 Universidad Autónoma de Nuevo León, Facultad de Ciencias Biológicas, Departamento de Microbiología e Inmunología, LMYF, Unidad de Manipulación Genética, Monterrey, Nuevo León, Mexico; 2 Tecnológico Nacional de México/Instituto Tecnológico de Durango, Departamento de Ingeniería Química y Bioquímica, Laboratorio Nacional CONAHCYT para la Evaluación de Productos Bióticos LaNAEPBi, Durango, Mexico

**Keywords:** fungal endosymbiosis, fungal-bacterial interactions, host-microbe coevolution, intracellular symbionts, progressive endosymbiosis

## Abstract

Endosymbiotic interactions in fungi are fundamental to ecological and evolutionary processes; however, despite their potential to elucidate key mechanisms of biological integration, they remain insufficiently explored. This review provides a comprehensive synthesis of the available evidence on these associations across major fungal lineages, encompassing their composition, functional attributes, and breadth across diverse model systems. Additionally, the current state of artificial endosymbiosis is examined, together with its applications in multiple areas of biotechnology, spanning both natural and engineered systems. Finally, a framework of progressive endosymbiosis is proposed to describe the *continuum* of integration states of endosymbionts within fungal hosts. Together, these insights highlight the central role of fungal endosymbiosis in biological integration and underscore its value as a model for understanding evolutionary transitions and developing innovative biotechnological applications.

## 1 Introduction

The kingdom *Fungi* represents one of the most diverse lineages within the eukaryotic domain, comprising both unicellular and multicellular organisms that occur across a wide range of ecosystems (Stajich, 2017; Priest et al., 2020). Despite this diversity, fungal biology has historically been underestimated, even though fungi exhibit complex life cycles, specialized structures, and diverse intracellular components that underpin their ecological success (Kelliher et al., 2023).

In natural environments, fungi occur either as free-living entities or in diverse symbiotic associations; among these, endosymbiosis constitutes the most intimate form of interaction, in which one organism resides intracellularly within its host (Wernegreen, 2012). In recent years, growing evidence has demonstrated that fungal cells can harbor other microorganisms, predominantly bacteria (Robinson et al., 2021), referred to as endobacteria or bacterial endosymbionts (Lastovetsky and Guan, 2026). Furthermore, the presence of archaea, eukaryotes, and viruses has been documented, indicating that these systems represent complex biological entities with diverse functional implications (Araldi-Brondolo et al., 2017; Richter et al., 2025).

Fungal endosymbioses are widely distributed phenomena, arising from the progressive intensification of ecological interactions driven by interdependence and metabolic integration. These associations have been examined through microscopy-based approaches and molecular techniques (Morales et al., 2022; Kelliher et al., 2023), with the most substantial support observed in early-diverging lineages such as *Glomeromycota*, *Mucoromycota*, and *Mortierellomycota*. In contrast, the phyla *Ascomycota* and *Basidiomycota* remain comparatively understudied (Bonfante and Desirò, 2017; Pawlowska et al., 2018; Uehling et al., 2023; Wallner et al., 2026).

This review aims to provide a comprehensive understanding of endosymbiosis in the fungal kingdom, integrating evidence from both natural models and synthetic systems. The diversity of associations and underlying mechanisms is examined comparatively across major fungal lineages, encompassing both well-studied groups and those less represented, while recognizing their functional relevance and biotechnological potential across diverse sectors.

Recent developments in endosymbiosis engineering are also examined, and future perspectives are outlined to understand how microbial interactions can scale from ecological networks to integrated biological units, thereby laying the groundwork for a theory of progressive endosymbiosis in fungi with implications for evolution, ecology, and biotechnology.

## 2 Natural endosymbiosis as the basis of an evolutionary *continuum*

Endosymbiotic associations in fungi should not be interpreted as isolated events, but rather as the outcome of persistent ecological interactions that, under specific selective pressures, have evolved toward increasingly intimate intracellular relationships. In this context, endosymbiosis can be conceptualized as a *continuum* ranging from facultative associations to highly integrated and interdependent systems, sustained by metabolic exchange and molecular communication between partners.

Current knowledge across different fungal lineages indicates that these interactions have arisen independently throughout evolution, giving rise to a remarkable diversity of symbiotic strategies. Despite this variability, recurring patterns can be identified that point to convergent mechanisms of functional integration.

**Figure 1:**
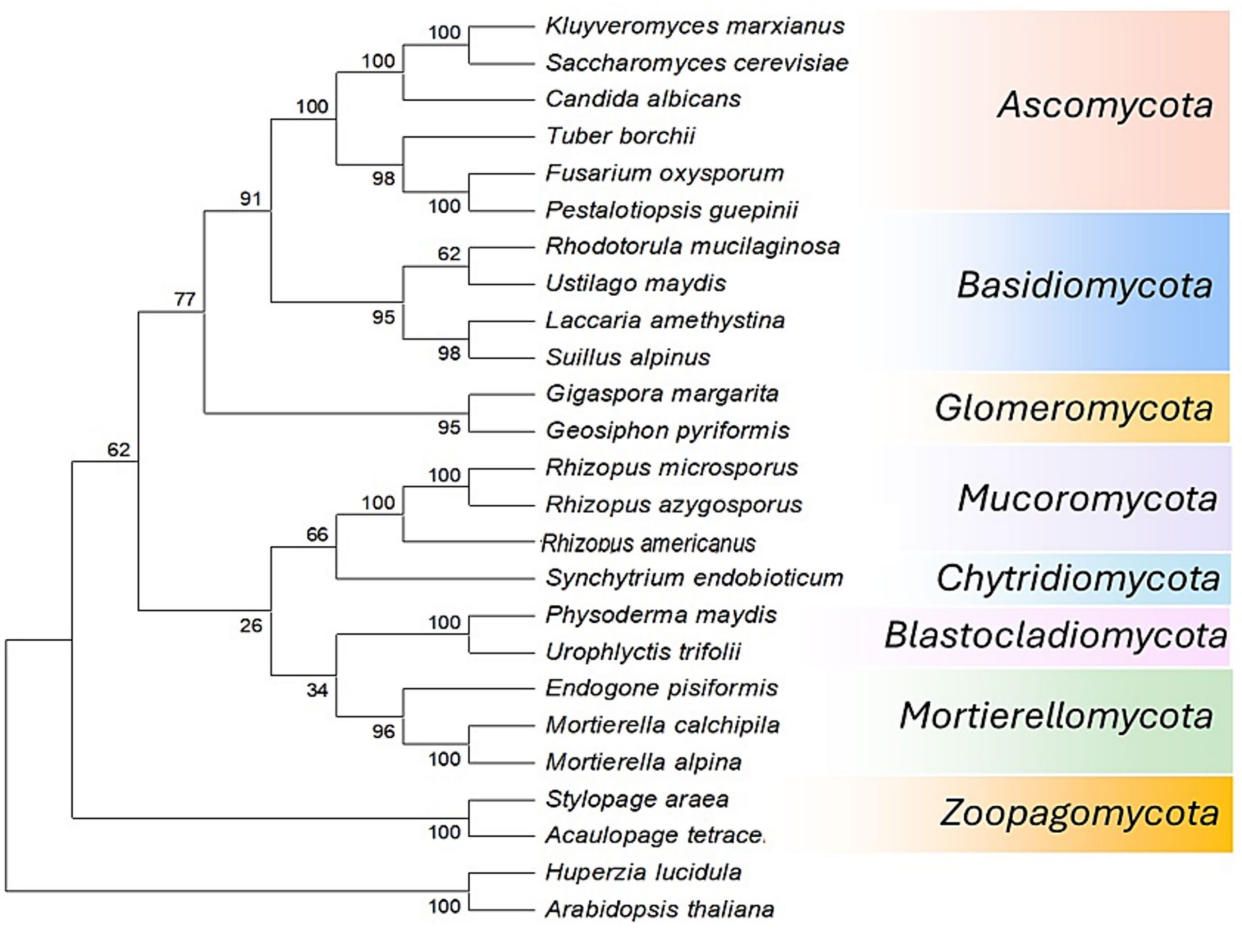
Phylogenetic tree of the principal phyla within the kingdom *Fungi*. Phylogenetic tree based on SSU (18S rRNA) gene sequences, illustrating the evolutionary relationships among representatives of the principal fungal phyla. Reference sequences were retrieved from the SILVA database (https://www.arb-silva.de/). The tree was inferred using the Maximum Likelihood method under the Tamura-Nei evolutionary model. Node support was assessed through bootstrap analysis with 1,000 replicates, and support values are indicated at the nodes. Representative species from each lineage were included, along with *Huperzia lucidula* and *Arabidopsis thaliana* as outgroups for tree rooting.

To frame this diversity within an evolutionary context, Figure 1 presents a phylogenetic tree of the principal phyla within the kingdom *Fungi*, considering *Glomeromycota*, *Mucoromycota*, and *Mortierellomycota* as independent phyla within the subkingdom *Mucoromyceta* (Zhao et al., 2023; Wijayawardene et al., 2024). This representation allows visualization of the distribution of fungal lineages and establishes a foundation for the comparative analysis of the endosymbiotic associations reported across them.

Within this structure, fungal endosymbiotic associations are examined from a chronological perspective, from the earliest reports to the most recent studies. Emphasis is placed on both their diversity and the evolution of methodological approaches, ranging from microscopy-based observations to molecular and genomic tools that have enabled the characterization of their functionality and ecological relevance, thereby providing the basis for proposing a trajectory of progressive evolutionary integration.

### 2.1 Early reports of fungal endosymbiosis: *Glomeromycota* as an ancestral model

#### 2.1.1 Geosiphon pyriformis

One of the earliest documented endosymbiotic systems in fungi corresponds to the association between *G. pyriformis* and cyanobacteria of the genus *Nostoc*, regarded as an early model of functional integration between phylogenetically distant organisms. The intracellular observation of *Nostoc* spp. cells within vesicles of *G. pyriformis* led to the hypothesis that both partners operate as a single functional unit, described as a macrosymbiont (von Wettstein, 1915; Knapp, 1933), in which the cyanobacterium may account for the host’s potential nitrogen-fixing capacity, supported by the detection of nitrogenase activity (Kluge et al., 1992). The genome of *G. pyriformis* contains genes indicative of a potential bacterial origin, including selenium-binding proteins and molybdenum cofactor transporters (Malar et al., 2021). In contrast, *Nostoc punctiforme* exhibits a genome size of 9.9 Mb, which is larger than that of free-living species, and displays a higher proportion of mobile genetic elements, including insertion-excision systems. This genomic context is accompanied by the presence of a *Mollicutes*/*Mycoplasma*-related endobacteria (*MRE*) population of approximately 721 kb, which shares only 3.3% of core genes with other *MRE* genomes reported in arbuscular mycorrhizal fungi (AMF) (Sorwar et al., 2024).

#### 2.1.2 Gigaspora margarita

The first consolidated model for the study of endosymbiotic microorganisms in fungi was *G. margarita*, established through the application of polymerase chain reaction (PCR) targeting the 16S rRNA gene. This approach enabled the demonstration of a viable and quantifiable bacterial population, vertically transmitted across all generations of fungal spores, initially assigned to the genus *Burkholderia* (Bianciotto et al., 1996; Bianciotto et al., 2004; Salvioli et al., 2008) and currently classified as *Candidatus* Glomeribacter gigasporarum (*Ca*Gg) (Bianciotto et al., 2003), within the *Burkholderia*-related endobacteria (*BRE*) group. This association represents an endosymbiotic relationship with *Glomeromycota* dating back at least 400 million years (Mondo et al., 2012). In addition, the co-occurrence of viral sequence populations has also been confirmed (Turina et al., 2018).

Efforts to isolate *Ca*Gg from its host have revealed genomes consisting of a chromosome ranging from 1.4 to 1.72 Mb and three plasmids. Genomic analyses indicate a reliance on host-derived carbon, phosphorus, and nitrogen, along with the expression of secretion systems (Jargeat et al., 2004; Ghignone et al., 2012). Independently of cultivation efforts, a chromosome of approximately 2.0 Mb was characterized, containing abundant mobile genetic elements and biosynthetic capacity for cofactor production, as well as two plasmids harboring genes associated with defense and signal transduction. Furthermore, the presence of *Ca*Gg influences the regulation of multiple transposable elements within the host genome (Mugambi et al., 2026).

From a functional perspective, the elimination of the endosymbiont population in *G. margarita* resulted in cell wall thickening and a reduced abundance of lipid and protein bodies (Lumini et al., 2007), a phenotype further supported by metabolic alterations and the induction of heat shock proteins under stress conditions (Salvioli et al., 2010). The association with *Ca*Gg in *G. margarita* is linked to differential expression of genes and proteins involved in chitin metabolism, iron and phosphate transport, phosphorylation processes, calcium-mediated signal transduction, regulation of reactive oxygen species (ROS), and peptidase activity (Salvioli et al., 2016; Vannini et al., 2016; Venice et al., 2020), as well as the expression of toxin-antitoxin systems encoded by *Ca*Gg (Salvioli di Fossalunga et al., 2017). In parallel, advanced defense-related responses have been described, including the involvement of mitogen-activated protein kinases (MAPKs) and mechanisms linked to DNA and chromatin remodeling under oxidative stress (Venice et al., 2017), together with an increased abundance of metabolites associated with the tricarboxylic acid cycle, the pentose phosphate pathway, antioxidant systems, and melanin biosynthesis (Dearth et al., 2018).

These observations highlight the plasticity of the endosymbiotic system and its ecological competence, reflected in its capacity to operate at a higher level of interaction. This is evidenced by the upregulation of the inorganic phosphate transporter gene in *Solanum lycopersicum* (Chialva et al., 2020) and modifications in plastidial lipid metabolism in *Lotus japonicus* (Venice et al., 2021), providing key insights into tripartite interactions involving fungi, endosymbionts, and plants.

Importantly, *MRE* has been reported, later more precisely identified as *Candidatus* Moeniiplasma glomeromycotorum (*Ca*Mg) (Naito et al., 2017). This endosymbiont exhibits a relative abundance 2.59 to 3.06 times higher than that of *Ca*Gg, representing the first report of a fungal microbiome (Desirò et al., 2014). This finding is consistent with the lower incidence of *Ca*Gg compared to *Ca*Mg across spores from different families. Environmental and host-related factors, including soil nutrient levels, plant density, and host genotype, have been identified as key determinants influencing the distribution of *Ca*Mg (Lastovetsky et al., 2018; Lastovetsky et al., 2024), which appears to exert a greater impact on carbon allocation than *Ca*Gg in *G. margarita* (Kuga et al., 2021).

#### 2.1.3 Other studies in *Glomeromycota*

Several AMF lineages, including *Glomus* spp., *Funneliformis* spp., *Rhizophagus* spp., and *Racocetra* spp., have revealed *MRE* populations (Naumann et al., 2010; Desirò et al., 2013; Agnolucci et al., 2015; Savary et al., 2021; Grassi et al., 2026), together with a higher degree of co-phylogenetic congruence within *Gigasporaceae* (Longley et al., 2024). The first genomic structure, obtained from *Dentiscutata heterogama*, showed a compact architecture of approximately 713 kb, including proteins with homology to fungal counterparts, lacking genes involved in cell wall biosynthesis, and exhibiting a markedly reduced metabolic capacity characterized by a limited number of nutrient transporters (Torres-Cortés et al., 2015). Similarly, other representatives lack biosynthetic pathways for purines and pyrimidines (Sun et al., 2019).

The complexity and adaptive capacity of *MRE* across multiple hosts are consistent with mutation accumulation and with approximately 5% of genes acquired through horizontal gene transfer (Naito et al., 2015; Toomer et al., 2015). In this context, these populations have been proposed to escape Müller’s ratchet, given their vertical transmission coupled with a high rate of molecular evolution, an abundance of pseudogenes, and an elevated degree of recombination facilitated by mobile genetic elements. These processes are thought to contribute to genomic plasticity and the purging of mildly deleterious mutations, thereby maintaining genetically diverse and stable populations (Naito and Pawlowska, 2015, 2016).

### 2.2 Functional consolidation of fungal endosymbiosis: representative systems in *Mucoromycota*

#### 2.2.1 Rhizopus microsporus

The assumption that *R. microsporus* was responsible for the biosynthesis of rhizoxin, the causal agent of rice seedling blight, was overturned following the intracellular identification of *Burkholderia rhizoxinica*, currently classified as *Mycetohabitans rhizoxinica* (Estrada-de Los Santos et al., 2018), as the toxin producer, as demonstrated through pure culture assays (Partida-Martinez and Hertweck, 2005; Partida-Martinez et al., 2007a). In this system, the macrolide undergoes a second epoxidation mediated by the host, indicating a synergistic biosynthetic process (Scherlach et al., 2012). Other toxins have been described, such as rhizonin, which is biosynthesized and modified exclusively by *Mycetohabitans endofungorum*, exhibiting production levels comparable to those observed during intracellular interaction with *R. microsporus* (Partida-Martinez et al., 2007b; Ehinger et al., 2023).

The genomic composition of *M. rhizoxinica* represents the first characterized genome of a cultivable fungal endosymbiont, comprising approximately 3.75 Mb distributed across a chromosome and two plasmids. This analysis revealed the occurrence of primary metabolic pathways, exopolysaccharide biosynthesis, toxin-antitoxin systems, abundant insertion elements, cofactor biosynthesis, membrane transport systems, and a limited number of transcriptional regulators (Lackner et al., 2011a; Lackner et al., 2011b; Uzum et al., 2015), together with a potential for secondary metabolism, including endopyrroles and cyclopeptides, identified across different isolates (Niehs et al., 2018a; Niehs et al., 2019). Comparative analyses with *Mycetohabitans* spp. indicate genome reduction accompanied by an expansion of transposable elements and pseudogenes, consistent with a transition from facultative to obligate endosymbiosis (Abbot et al., 2025).

The bacterial endosymbionts are broadly distributed and have been reported across diverse geographic locations (Lackner et al., 2009). The interaction has been characterized as mutualistic, as vegetative reproduction of *R. microsporus* does not occur in the absence of *M. endofungorum*, suggesting the loss of endogenous factors required for sporulation (Partida-Martínez et al., 2007c). Within this framework, deazaflavin, genes encoding the type III secretion system (T3SS), and transcription activator-like (TAL) effectors secreted through this system in *M. rhizoxinica* have been shown to be essential for the induction of sporulation (Lackner et al., 2011c; Richter et al., 2023a; Richter et al., 2024). Moreover, these effectors enhance membrane stress tolerance in the host (Carter et al., 2020), and their diversity suggests multiple mechanisms contributing to the interaction (Carpenter et al., 2024).

The functional scope of endosymbionts in *R. microsporus* extends to mating processes, through the regulation of *ras2-1* expression and candidate genes associated with the perception of trisporic acid pheromones, interpreted as adaptive changes that have reinforced the symbiotic relationship (Mondo et al., 2017). Additionally, two Narnaviruses capable of replication and vertical transmission within the host have been identified. Although they reduce asexual reproduction, they act as conditional mutualists alongside the endosymbiotic population in maintaining sexual reproduction (Espino-Vázquez et al., 2020), with *in silico* predictions further suggesting alterations in nucleotide and lipid metabolism in aposymbiotic *R. microsporus* (Valadez-Cano et al., 2024).

In relation to bacterial colonization in *R. microsporus*, several factors have been described, including the galactofuranosyl O-antigen (Leone et al., 2010), horlicin A, involved in surface tension reduction and biofilm formation (Niehs et al., 2018b), the *habA* gene, associated with the habitasporin biosynthetic cluster (Niehs et al., 2022), and TAL1, which ensures the intracellular persistence of *M. rhizoxinica* by preventing its entrapment through fungal septation (Richter et al., 2023b). The type II secretion system (T2SS) has been proposed as essential for the active invasion of hyphae through the secretion of chitinases, consistent with a model of site-specific cell wall lysis as part of an endocytosis-related mechanism (Moebius et al., 2014). Complementary processes documented during physical interaction between partners include the production of exopolysaccharides, lipid metabolism, cytoskeletal rearrangement, the high-osmolarity glycerol (HOG) signaling pathway, MAPK cascades, cyclic adenosine monophosphate (cAMP) signaling, and antioxidant defense responses (Lastovetsky et al., 2016; Lastovetsky et al., 2020).

Interest in these endosymbionts has extended to the food sector following the identification of *Mycetohabitans* spp. in *R. microsporus* isolated from agricultural soils and fermentation processes, together with the detection of rhizoxin derivatives (Rohm et al., 2010; Dolatabadi et al., 2016; Cabrera-Rangel et al., 2022). Other species, such as *Rhizopus oryzae*, harbor bacteria including *Serratia marcescens*, *Pseudomonas fluorescens*, and *Klebsiella pneumoniae* (Birol and Gunyar, 2021), as well as *Pandoraea sputorum* and *M. endofungorum*, whose genomes encode toxin-related genes (Liu et al., 2024). These findings underscore the importance of sanitary monitoring of fungi in food-related contexts and highlight the need for studies assessing their potential risk to human health.

Although clinical isolates of *R. microsporus* exhibit fewer endosymbiotic associations (Stairs et al., 2026), earlier reports described rhizoxin-producing *Mycetohabitans* spp. without apparent pathological correlations (Ibrahim et al., 2008; Gee et al., 2011). More recently, cases involving immunocompromised patients with bacteremia caused by *M. rhizoxinica* and *M. endofungorum*, alongside invasion by *R. microsporus*, have been documented, in which early detection of endosymbionts may represent a preliminary indicator preceding the diagnosis of fungal coinfection (Yang et al., 2022; Orbea et al., 2025; Tyler et al., 2026). Cases of rhinocerebral mucormycosis with severe progression in the absence of classical risk factors have also been reported (Tansarli et al., 2023). Transcriptomic profiles of these interactions indicate the activation of defense responses during macrophage-mediated phagocytosis (Sephton-Clark et al., 2020); however, further studies will be required to better elucidate the implications of this endosymbiosis for human health and to explore the potential for developing endosymbiont-targeted therapeutic strategies (Itabangi et al., 2022).

#### 2.2.2 Other studies in *Mucoromycota*

*Endogone* sp. is notable as one of the earliest formal reports describing the observation of bacteria-like bodies by electron microscopy (Mosse, 1970), with *MRE* populations belonging to a clade distinct from those associated with *Glomeromycota* (Desirò et al., 2015), a pattern that has also been observed in *Mucor* sp. (Okrasińska et al., 2021). Likewise, the occurrence of *BRE* in *Umbelopsis* sp. indicates the presence of free-living *Paraburkholderia* strains (Okrasińska et al., 2021), including a cultivable representative described in *Saksenaea boninensis* (Takashima et al., 2023).

Other endosymbionts have been described, such as *S. marcescens* in *Mucor irregularis* (Hazarika et al., 2020) and *Paenibacillus* sp., which exhibits a notable genomic capacity for carbon assimilation and is associated with *Thamnidium elegans* (Muszewska et al., 2021).

### 2.3 *Mortierellomycota* as a model of genome reduction and endosymbiotic specialization

#### 2.3.1 Linnemannia elongata

The emergence of this fungal model can be traced to the identification of an intracellular betaproteobacterium phylogenetically related to *Ca*Gg and *Burkholderia* spp. (Sato et al., 2010), later classified as *Mycoavidus cysteinexigens* (Ohshima et al., 2016), with its endosymbiotic association estimated to have originated more than 350 million years ago (Uehling et al., 2017).

The genome of *M. cysteinexigens*, comprising 2.79 Mb, encodes proteins involved in essential cofactor biosynthesis and insecticidal toxin production; however, it lacks the pathway for cysteine biosynthesis as well as components of T2SS (Fujimura et al., 2014; Sharmin et al., 2018). Furthermore, *MRE* has been identified, featuring a genome of approximately 326 kb, representing the smallest endofungal genome described to date, characterized by the reduction of genes associated with carbohydrate metabolism, membrane transport, homologous recombination, and mismatch repair (Longley et al., 2023).

Removal of the *M. cysteinexigens* population results in increased accumulation of fatty acids and reduced storage of carbohydrates, organic acids, and nitrogen-containing compounds in the host. By contrast, the profile of volatile organic compounds is more abundant in association with the endosymbionts, including alcohols, aldehydes, ketones, and furans that may influence microbial interactions (Uehling et al., 2017). Under nitrogen-limited conditions, *L. elongata* associated with *M. cysteinexigens* exhibits an increased relative abundance of bacterial proteins linked to active proliferation, accompanied by a reduction in fungal intermediates involved in chitin biosynthesis. The endosymbiont appears to utilize malate, amino acids, dipeptides, spermidine, and *γ*-aminobutyric acid under stress conditions, suggesting a multimodal and interconnected metabolic regulation in response to environmental factors (Li et al., 2017).

#### 2.3.2 Other studies in *Mortierellomycota*

Diversity within *Mortierella* spp. is extensive and includes *BRE* with morphology resembling that of *M. cysteinexigens*, grouped into distinct subclades (Okrasińska et al., 2021; Amses et al., 2023), whose occurrence has been associated with sexual infertility (Takashima et al., 2020). Concurrent detection of *MRE* and *Mycoavidus* sp. has also been reported in certain hosts, together with observations of reduced fungal biomass relative to *MRE*-cured counterparts (Desirò et al., 2018).

From a genomic perspective, species of *Mycoavidus* have been isolated from the genera *Actinomortierella*, *Podila*, *Linnemannia*, and *Mortierella*, with genome sizes ranging from 2.1 to 3.2 Mbp and characterized by the loss of biosynthetic pathways such as those for histidine and threonine, in agreement with the phylogenetic clustering of the strains (Amses et al., 2023). Similarly, isolates obtained from *Mortierella parvispora*, exhibiting a smaller genome size of approximately 1.88 Mb, suggest a non-degenerative evolutionary trajectory attributed to repeated insertion–deletion events involving prophages and transposons. Comparisons with free-living relatives indicate that these elements may define core genetic components of a minimal cultivable genome (Guo et al., 2020).

Additional bacteria, including *Castellaniella defragrans* and *Cryobacterium* sp., identified in *Mortierella alpina*, produce N-acylhomoserine lactones, quorum-sensing molecules that may participate in the symbiosis (Kai et al., 2012). Potential horizontal gene transfer of endobacterial origin linked to fungal metabolites has also been inferred (Wurlitzer et al., 2021).

### 2.4 Evidence of endosymbiotic associations in *Zoopagomycota*

Although studies in this phylum remain limited, aphid-pathogenic fungi such as *Pandora* spp. indicate a higher frequency of *Gammaproteobacteria* and their transmission from hyphae to conidia, together with increased endosymbiotic diversity, potentially associated with enhanced fungal virulence following prior interaction with aphid hosts (Chen et al., 2016). In parallel, sequencing of *Acaulopage tetraceros* and *Stylopage hadra* revealed sequences affiliated with *Chitinophaga* sp. and *M. cysteinexigens*, respectively (Davis et al., 2019). Further, deeper sequencing approaches have enabled the assembly of genomes from *Chryseobacterium* sp., *Pantoea vagans*, *Pseudomonas* sp., and *Lactococcus lactis* from the metagenome of *Massospora cicadina* (Ettinger et al., 2022).

### 2.5 Complexity and versatility of endosymbiotic associations in *Ascomycota*

#### 2.5.1 *Candida* spp.

The intracellular localization of *Helicobacter pylori* in *Candida* spp. and within their vacuoles, based on oral, gastric, and fecal samples, combined with microscopic, molecular, and immunofluorescence approaches, has established this system as a model of endosymbiosis in clinical contexts (Siavoshi et al., 1998; Siavoshi et al., 2005; Salmanian et al., 2008; Saniee et al., 2013a; Saniee et al., 2013b; Saniee and Siavoshi, 2015; Matamala-Valdés et al., 2018; Sánchez-Alonzo et al., 2020a; Heydari et al., 2022; Sun et al., 2026). Nevertheless, additional bacterial taxa have been identified across diverse ecological niches, including *Staphylococcus* spp., *Microbacterium* sp., and *Leptolyngbya boryana* (Kang et al., 2009; Siavoshi et al., 2018; Tavakolian et al., 2019; Ebrahimi et al., 2020; Siavoshi et al., 2020; Heydari et al., 2022), as well as archaea belonging to the *Asgard* superphylum (Jagadeeshwari et al., 2023).

In general, *Candida* spp. and their vacuoles have been proposed as a potential reservoir for *H. pylori* (Siavoshi and Saniee, 2014; Siavoshi et al., 2019), as multiple abiotic stress factors, including acidic pH, exposure to antibiotics such as amoxicillin, nutrient limitation, elevated temperatures, anaerobic conditions, and specific strain combinations, promote increased internalization (Sánchez-Alonzo et al., 2020b, 2021a, 2021b, 2021c, 2022; Hiengrach et al., 2022). Regarding antimicrobial treatments, amphotericin B has been shown to release *Staphylococcus hominis* and *Staphylococcus haemolyticus*, although only in a small proportion of *Candida* spp. (Tavakolian et al., 2018), whereas a cocktail composed of amoxicillin, ciprofloxacin, rifampicin, and metronidazole failed to eliminate *H. pylori*, *Staphylococcus*, *Nocardia*, or *Cyanobacteria* in *Candida tropicalis* (Hatefi et al., 2025).

A major concern arises from the detection of *H. pylori* and genes associated with its virulence in *Candida* strains isolated from vaginal discharge, particularly in pregnant women (Siavoshi et al., 2013; Sánchez-Alonzo et al., 2021d; Yang et al., 2023; Yang et al., 2024). Experimental evidence in murine models indicates vertical transmission to offspring (Xu et al., 2026), representing a potential risk of neonatal infection during childbirth.

#### 2.5.2 *Fusarium* spp.

Endosymbionts associated with this genus exhibit considerable diversity (Li et al., 2010; Shaffer et al., 2016). Among the reported effects, *Enterobacter* sp. has been shown to influence macroconidia production, as well as to enhance fumonisin biosynthesis and disease severity in soybean seedlings infected with *Fusarium fujikuroi* (Obasa et al., 2020) and contribute to the virulence of *Fusarium oxysporum* in banana seedlings (Rahayu and Maulana, 2020). Consistently, association with *Stenotrophomonas maltophilia* in *Fusarium graminearum* has been linked to increased lesion development in coleoptiles of wheat seedlings (Ali et al., 2022).

Nevertheless, beneficial effects have also been described, including enhanced growth of *Fusarium keratoplasticum* in association with *Chitinophaga* sp. across different substrates (Shaffer et al., 2017), together with the promotion of plant growth and phytohormone production (Cheng et al., 2022; Fang et al., 2023).

The endomicrobial composition of different *Fusarium* isolates, assessed through co-culture approaches, shows stimulation in response to hyphosphere-associated bacteria, which may contribute to the maintenance of core taxa such as *Buttiauxella*, *Erwinia*, and *Citrobacter*. In this context, variability among strains and environmental conditions is critical for understanding the dynamics of these interactions in natural settings (Lupini et al., 2023; Lupini et al., 2025).

#### 2.5.3 *Pestalotiopsis* sp.

Foliar endophytes have revealed both uncultivable bacterial members and *Luteibacter mycovicinus*, whose interaction with its host has been associated with enhanced production of indole-3-acetic acid (IAA) (Hoffman and Arnold, 2010; Hoffman et al., 2013; Arendt et al., 2016; Baltrus et al., 2017; Baltrus et al., 2024). As part of the establishment of this symbiosis, the bacterial partner upregulates genes associated with the type VI secretion system (T6SS), whereas *Pestalotiopsis* sp. exhibits metabolic activity linked to methionine, reflecting the dependence of *L. mycovicinus* on the host for sulfur acquisition (Baltrus and Arnold, 2017; Shaffer et al., 2022).

#### 2.5.4 Other studies in *Ascomycota*

*Tuber borchii* represents one of the earliest models investigated using fluorescence *in situ* hybridization (FISH) and quantification of members of the former *Cytophaga-Flexibacter-Bacteroides* phylum (Barbieri et al., 2000; Barbieri et al., 2002). Among nematophagous fungi, *Arthrobotrys musiformis* harbors a diverse endomicrobial community, with potential contributions to nitrogen metabolism, along with bacterial isolates capable of enhancing nematode trap formation (Zheng et al., 2024), whereas in *Esteya vermicola*, associations with *Stutzerimonas stutzeri* and *Candidatus* Phyllobacterium have been documented (Wang et al., 2017; Wang et al., 2022). In contrast, entomopathogenic fungi such as *Metarhizium* spp. have been reported to host *Pelomonas puraquae* and *Bacillus subtilis*, whose association has been linked to reduced host virulence (Ying et al., 2022; Mora-Acebedo et al., 2025).

Within current research approaches, a major objective lies in deciphering the core microbiome. Several representatives of *Sordariomycetes* share bacterial communities characterized by *Bacillales*, *Burkholderiales*, *Enterobacterales*, *Hyphomicrobiales*, and *Pseudomonadales*. A potential specialization has been proposed for *Amphisphaeriales*, supported by the identification of *Moraxellales*, *Sphingomonadales*, and *Streptosporangiaceae*, whereas in *Glomerellales*, *Enterobacterales*, *Hyphomicrobiales*, and *Micrococcales* are prominent (Escudero-Leyva et al., 2025). Clinical isolates of *Aspergillus fumigatus* exhibit associations with *Ralstonia*, the *Burkholderia*-*Caballeronia*-*Paraburkholderia* (BCP) group, and *Methylobacterium*-*Methylorubrum*, together with a potential contribution of *Bryobacter* to increased fungal virulence (Piontkivska et al., 2025). These studies highlight the importance of further characterizing endomicrobial communities to elucidate host-specific associations, ecological niche effects, and additional factors that remain uncharacterized.

Regarding yeast systems, bacterial isolation has been facilitated through the induction of stress conditions, including cellular aging, nutrient deprivation, exposure to antimicrobials, and mechanical perturbation. In *Candida* spp. and *Saccharomyces* spp., bacterial taxa such as *Arthrobacter* sp., *Cellulomonas* sp., *Staphylococcus xylosus*, and *S. haemolyticus* have been successfully recovered (Heydari et al., 2020). Also, *Nocardia* sp. has been isolated from *Coniochaeta polymorpha* (Heydari et al., 2021), while in *Kluyveromyces marxianus*, association with *Bacillus tequilensis* has been reported, enabling nitrogen fixation (Mares-Rodriguez et al., 2023). In parallel, 16S rRNA gene sequencing of *Candida* spp. and *Pichia* spp. from environmental samples identified *Staphylococcus* as the most abundant genus, although overall bacterial diversity was heterogeneous (Indu et al., 2021). By contrast, *Wickerhamomyces anomalus* strains isolated from mosquitoes exhibited a higher abundance of *Stenotrophomonas*, without a clear trend related to the isolation niche (Cappelli et al., 2023). Additionally, wild yeasts display higher abundances of *Escherichia* and *Comamonas*, with endomicrobial composition shaped by environmental conditions (Iturritxa et al., 2026).

### 2.6 Advanced functionality of endosymbiosis in *Basidiomycota* with implications for nutrition and development

#### 2.6.1 Piriformospora indica

This plant-associated fungus harbors *Rhizobium radiobacter*, whose morphology differs between its intracellular state and its cultivable form, while maintaining a genome size comparable to that of free-living strains. Although the endosymbiont has not been successfully eliminated through antibiotic treatments, a reduction in its population compromises host fitness and sporulation (Sharma et al., 2008; Glaeser et al., 2016; Guo et al., 2017; Glaeser et al., 2017). Moreover, N-acyl homoserine lactones have been identified as key factors in the promotion of plant growth (Alabid et al., 2020).

#### 2.6.2 Ustilago maydis

In *U. maydis*, a confirmed capacity for nitrogen fixation was first reported, attributed to intracellular association with *Bacillus pumilus*, suggesting a functional exchange in which the host supplies carbon while the endosymbiont provides ammonium (Ruiz-Herrera et al., 2015). All wild strains isolated from naturally infected maize plants contain endobacteria; however, not all exhibit nitrogen-fixing capacity, indicating a heterogeneous composition (Pérez-Rodríguez et al., 2021). Other species, such as *Klebsiella michiganensis*, have also been found to be enriched, exhibiting nitrogen-fixing capability alongside the identification of *nif* genes in their genome (Liang et al., 2023). Within this framework, future research may focus on determining how endosymbionts influence the pathogenicity of this and other models, including *Fusarium* spp. (Moses and Carter, 2025).

#### 2.6.3 Rhodotorula mucilaginosa

Despite lacking a functional pathway for nitrate assimilation, *R. mucilaginosa* can grow in nitrogen-free media (Sen et al., 2019), a phenomenon attributed to endosymbiotic association with *S. stutzeri*, which confers nitrogen-fixing ability. In conjunction with the host, this interaction enhances the nutritional status of *Oryza sativa* seedlings (Paul et al., 2020). Metagenomic analyses have revealed that the yeast harbors a complex endomicrobial community, with a notable abundance of *S. stutzeri* and *Bacillus velezensis* under nitrogen-limited conditions, together with the identification of genes associated with nitrogen fixation and dissimilatory nitrate reduction (Nag et al., 2024). However, *nif* genes have not been detected in the cultivable form of *B. velezensis* (Nag and Seal, 2024), underscoring the need for further investigation into nitrogen metabolism under this symbiotic interaction.

#### 2.6.4 Other studies in *Basidiomycota*

Early approaches focused on the detection of endomicrobial communities in ectomycorrhizae, including representatives such as *Laccaria* spp. and *Suillus* spp. (Bertaux et al., 2003; Bertaux et al., 2005; Izumi et al., 2006; Hermawan et al., 2023). Subsequent investigations of fruiting bodies have provided relevant structural insights, revealing communities dominated by *Pseudomonas*, *Sphingomonas*, and members of the BCP group (Pent et al., 2020), whose representatives have also been isolated alongside *Bacillus* spp. and *Priestia* spp. across a range of fungal hosts (Efimenko et al., 2016; Aslani et al., 2018; Bai et al., 2021; Zhang et al., 2023; Paul et al., 2023; Paul et al., 2025).

Although the functional impact of endosymbionts has been less extensively explored, *Enterobacter* sp. has been reported to increase the virulence of *Rhizoctonia solani* in bentgrass (Obasa et al., 2017; Zhang et al., 2024). In *Guyanagaster necrorhizus*, whose nitrogen-fixing capacity has been associated with members of *Enterobacteriaceae*, an anaerobic representative has been described whose abundance increases with sporocarp maturation and correlates with enhanced acetylene reduction (Koch et al., 2021). A comparable pattern of endomicrobial variation across developmental stages has also been observed in *Cantharellus cibarius* (Gohar et al., 2020).

### 2.7 Knowledge gaps in fungal endosymbiosis in *Blastocladiomycota* and *Chytridiomycota*

To the best of current knowledge, and consistent with previous reports (Wallner et al., 2026), endobacterial associations have not been specifically described in either of these fungal phyla. This lack of information highlights the importance of investigating these groups within an evolutionary context, as well as determining whether they also harbor *MRE* populations, which would allow assessment of whether this trajectory is conserved in non-*Dikarya* fungi.

## 3 Artificial endosymbiosis as a model to explore the evolution of intracellular systems

Efforts to reintroduce endosymbionts into their native hosts, particularly in *R. microsporus* (Partida-Martínez et al., 2007c) and *P. indica* (Baltrus et al., 2018), have not only provided valuable insights into the mechanisms underlying internalization, colonization, and symbiosis, but have also laid the groundwork for artificial endosymbiosis, representing the establishment of endosymbionts within host cells in which such associations do not naturally occur, with objectives ranging from the study of syntrophic interactions and tolerance to the evaluation of evolutionary processes aimed at emulating organelle-forming associations in eukaryotes (Meaney et al., 2020; Puri et al., 2021).

### 3.1 Saccharomyces cerevisiae

Endosymbiosis engineering approaches have enabled experimental examination of the endosymbiotic theory of mitochondria. The introduction of a thiamine-auxotrophic *Escherichia coli* strain into the cytosol of respiration-deficient *S. cerevisiae* resulted in sustained persistence for up to 40 generations, demonstrating a mutually dependent relationship. This phenomenon was accompanied by the expression of soluble NSF attachment protein receptor (SNARE) proteins, presumably involved in evading lysosomal degradation (Mehta et al., 2018; Mehta et al., 2019). Comparable systems based on auxotrophy have further enabled the identification of *E. coli* genes whose modification across multiple passages involved transposon insertions at specific loci, associated with intracellular persistence under osmotic stress conditions (Supekova et al., 2024).

The evolutionary trajectory of the chloroplast has also been explored through experimental platforms involving mutant strains of *Synechococcus elongatus* and *S. cerevisiae*, exhibiting sustained metabolic coupling for at least 20 generations. This association reflects a functional interdependence in which the cyanobacterium supplies ATP to the yeast, while the latter provides methionine to the endosymbiont (Cournoyer et al., 2022). In this context, engineered strains of *S. elongatus* expressing ATP/ADP transporters rely critically on ATP export to maintain symbiotic stability (De et al., 2024). Further expansion of the functional scope of these interactions has been achieved through the generation of *S. elongatus* mutants capable of secreting glucose, supplying ATP, and expressing SNARE proteins, which, in the presence of CO_2_, promote the propagation of mitochondrial mutants of *S. cerevisiae* in the absence of exogenous carbon sources (Gao et al., 2024).

*Wolbachia pipientis*, an obligate endosymbiotic bacterium of arthropods (Porter and Sullivan, 2023), has been experimentally introduced into *S. cerevisiae*, where infection over a 14-day period resulted in an increased number of mitochondria compared to aposymbiotic cells. This pattern was consistent with elevated oxidative phosphorylation activity, which may account for the premature death of the yeast (Uribe-Alvarez et al., 2019).

### 3.2 R. microsporus

Fluidic force microscopy enabled the introduction of *E. coli* as a non-endosymbiotic bacterium, which exhibited an unstable interaction within a host that naturally lacks endosymbionts. In contrast, *M. rhizoxinica*, as the native endosymbiont, led to the establishment of a functional symbiotic interaction, accompanied by rhizoxin production, suggesting the transfer of metabolic capabilities as an integral component of the system (Giger et al., 2024). However, *R. picketti* displayed a distinct behavior, characterized by greater colonization of fungal spores compared to *M. rhizoxinica*. This interaction was associated with the expression of genes involved in cell wall remodeling and the generation of ROS during early stages of colonization, followed by reduced regulation at later stages, a trend interpreted as part of host defense modulation and as a potential adaptive trajectory from antagonistic interactions toward commensal states (Gassler et al., 2025).

#### 3.2.1 Other studies on artificial endosymbiosis

Ralsolamycin produced by *Ralstonia solanacearum* acts as an inducer of chlamydospore formation in diverse fungi (Spraker et al., 2016). Its presence also facilitates the colonization of chlamydospores of *Aspergillus flavus* by free-living bacteria such as *E. coli*, *Pseudomonas aeruginosa*, and *Herbaspirillum seropedicae*. Notably, *P. aeruginosa* in its endofungal state enhances host fitness under starvation conditions. The timing of arrival of *P. aeruginosa* and *R. solanacearum* to chlamydospores is a critical determinant of dual colonization rates, highlighting the importance of temporal dynamics in shaping microbial community structure (Venkatesh et al., 2022). However, co-cultivation with *R. solanacearum* cells did not permit the incorporation of *E. coli* into *F. oxysporum*, indicating a host-dependent effect (Tsumori et al., 2023).

In a separate system, *Nannochloropsis oceanica* was successfully internalized and engaged in carbon and nitrogen exchange with *L. elongata*, with both symbionts remaining physiologically active for at least two months, representing the first report of an intracellular fungus-alga symbiosis (Du et al., 2019). Plate confrontation assays further enabled the introduction of *Pedobacter* sp. into *Morchella* sp., resulting in enhanced growth of endosymbiotic morels and increased tolerance to 4-coumaric acid compared to parental fungal strains (Zhang et al., 2025).

## 4 Biotechnological potential of fungal endosymbiosis

Endosymbiotic associations in fungi, beyond fulfilling ecological roles (Büttner et al., 2021, 2022; Richter et al., 2022), are increasingly recognized as an emerging axis in multiple areas of biotechnology. These applications encompass the synergistic use of symbiotic consortia as functional platforms, the direct exploitation of endosymbionts, and genome mining approaches aimed at the identification and production of natural products.

### 4.1 Agriculture

Following the identification of a putative phosphate transport operon and the expression of genes associated with nitrogen fixation by *Ca*Gg in *G. margarita*, its potential use as a biofertilizer was proposed (Ruiz-Lozano and Bonfante, 1999; Minerdi et al., 2001). Subsequent studies have described additional beneficial associations across different contexts, as summarized in Table 1.

**Table 1:** Applications of fungal endosymbiosis in agriculture.

Category	Endosymbiotic system	Description	Reference
Plant growth promotion	*Tylopilus neofelleus* and *Bacillus* sp.	Increased solubilization of chelated inorganic phosphorus and enhanced phosphorus uptake in *Pinus sylvestris*	Zhang et al. (2023)
	*Suillus grevillea* and *Cedecea lapagei*	Enhanced phytate mineralization and uptake in *Pinus massoniana*	Mei et al. (2024)
	*R. mucilaginosa*-*S. stutzeri*	Increased root and shoot length, as well as plant biomass, in *O. sativa*, accompanied by the regulation of genes involved in nitrogen metabolism and transport	Paul et al. (2020)
	*F. oxysporum*-*Klebsiella aerogenes*	Increased root and stem length in tomato seedlings, associated with IAA production	Cheng et al. (2022)
	*Fusarium acuminatum* and *B. subtilis*	Enhanced root and shoot growth in rice	Fang et al. (2023)
Biological control	*P. indica* and *R. radiobacter*	Induction of resistance in barley against infection by *Blumeria graminis*	Sharma et al. (2008)
	*Bacillus* spp. and *Sphingomonas paucimobilis* from *Cupressus sempervirens*	*In vitro* inhibition of various phytopathogenic fungi by volatile organic compounds and bacterial metabolites	Pakvaz and Soltani (2016)
	*Bacillus* spp. and *Pseudomonas* spp. from mushrooms	Reduction of brown spot symptoms and internal necrosis in *Agaricus bisporus*	Aslani et al. (2018)
Bioremediation	*Benniella erionia*-*MRE*	Improved tolerance to heavy metals and enhanced removal capacity	Lupini et al. (2022)
	*P. indica*-*Rhizobium* sp.	Reduced translocation of arsenic from soil to shoots in tomato, accompanied by growth promotion	Ahmad et al. (2022)

There is currently growing interest in understanding the functional roles of endosymbionts in AMF and their relationship with abiotic factors, such as drought stress tolerance in *Agave tequilana* (Chávez-González et al., 2024).

### 4.2 Clinical

Antibacterial and antifungal properties have been documented through various experimental approaches, including the study of bacterial isolates derived from fruiting bodies (Efimenko et al., 2016) and pigments such as prodigiosin produced by *S. marcescens*, associated with biofilm inhibition (Hazarika et al., 2021). Additionally, fungal metabolites such as spiromarmycin, identified in the marine strain *Spiromastix* sp. in association with its endosymbiont *Alcaligenes faecalis* (Shao et al., 2020), as well as compounds with antitubercular activity, have also been identified (Yao et al., 2026).

Among other effects, structural derivatives of rhizoxin have demonstrated antiproliferative properties (Scherlach et al., 2006), whereas polyketides derived from the association between *Aspergillus spelaeus* and *Sphingomonas echinoides* exhibit anti-inflammatory activity (Yang et al., 2025). From a pharmacological perspective, endolides produced by *Burkholderia contaminans*, isolated from *Sphingomonas leidyi*, show affinity for vasopressin and serotonin receptors (Almeida et al., 2016; Fisch et al., 2017; Almeida et al., 2018).

### 4.3 Industrial

Synthetic biology, supported by genomic and functional advances, is driving the development of systems capable of identifying and activating gene clusters responsible for natural products that remain uncharacterized (Wang et al., 2018; Niehs et al., 2020). In this regard, several research directions are being pursued, including the complementation of endosymbiotic cofactors for biocatalytic applications (Braga et al., 2019), the characterization of thermostable lasso peptides (Bratovanov et al., 2020; Fernandez et al., 2024), the exploration of microalgae and bacteria for lipid and biofuel production (Du et al., 2019; Liang et al., 2026), as well as the use of cyanobacteria in photosynthetic bioproduction systems in association with yeasts (De et al., 2024; Gao et al., 2024).

More recently, particular attention has been directed toward harnessing interconnected metabolic capabilities derived from endosymbiosis in yeast-based systems such as *S. cerevisiae*, where intracellular bacterial metabolic activity has been evidenced through the detection of signaling molecules (Tramice et al., 2025). Functional advantages are also evident in endosymbiotic interactions such as *K. marxianus*-*B. tequilensis*, in which ammonium production by the endosymbiont may enable enhanced biosynthesis of aromatic compounds, such as 2-phenylacetate, in the absence of exogenous nitrogen sources, without apparent toxicity during fermentation processes (Mares-Rodriguez et al., 2023; González-Lozano et al., 2025).

Exploration of this field is increasingly oriented toward understanding metabolic complementarity and the rational design of microbial consortia aimed at optimizing fermentation processes and the production of high-value molecules in the food, cosmetic, and fragrance industries, representing a biosafe and sustainable alternative that does not require direct genetic modification.

## 5 Progressive endosymbiosis theory: an integrative framework for the evolution of intracellular associations in fungi

**Figure 2:**
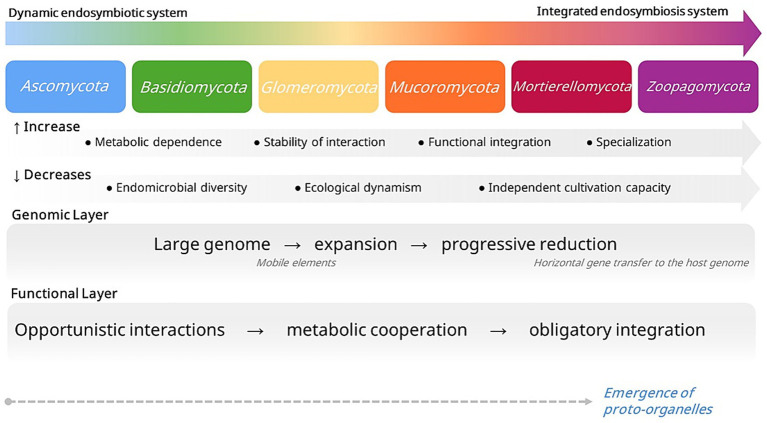
Model of progressive endosymbiosis in the fungal kingdom. An evolutionary *continuum* of fungal endosymbiotic interactions is proposed, spanning from dynamic, diverse, and environmentally dependent associations, such as those observed in *Ascomycota*, to highly specialized, stable, and metabolically integrated interactions in lineages such as *Zoopagomycota*. Along this gradient, intermediate states can be identified, characterized by the acquisition of specific functions (*Basidiomycota*), vertical transmission and the structuring of fungal microbiomes (*Glomeromycota*), as well as metabolic co-dependence and integration into biosynthetic pathways (*Mucoromycota*). At more advanced stages, a progressive reduction in genome size and near-complete dependence on the host are observed (*Mortierellomycota*), accompanied by loss of cultivability and functional specialization. Collectively, these trends suggest that fungal endosymbiosis evolves from open systems toward highly integrated associations, with the potential to represent early stages in the transition toward proto-organelles.

Insights from diverse fungal lineages suggest that endosymbiosis is not an isolated event, but rather a dynamic evolutionary process that transitions from highly flexible and diverse associations to more constrained, stable, and functionally integrated interactions. In this regard, we propose a theory of progressive endosymbiosis, in which the interactions observed in extant fungi represent distinct states along an evolutionary *continuum*, as illustrated in Figure 2.

Within this framework, more recently derived systems, such as those observed in *Ascomycota*, reflect early stages of the process, characterized by high endomicrobial diversity, transient and environmentally dependent associations, and a notable plasticity in the internalization of diverse bacterial groups. In genera such as *Candida* and *Fusarium*, complex intracellular communities have been documented, including bacteria and even archaea, with variable effects ranging from increased virulence to metabolic and ecological benefits. These associations may be induced by stress conditions (Castro-Seriche et al., 2021) and, in many cases, allow the isolation of cultivable bacteria, suggesting interactions that remain incompletely stabilized. Fungal vacuoles act as intracellular reservoirs, facilitating the coexistence of multiple taxa and reflecting a highly dynamic system in which metabolic cooperation is shaped by ecological context (Muszewska et al., 2021; Muradova et al., 2026).

In *Basidiomycota*, these associations exhibit an intermediate degree of stabilization. In models such as *U. maydis* and *R. mucilaginosa*, endosymbionts contribute clearly defined functions, including nitrogen fixation and plant growth promotion (Ruiz-Herrera et al., 2015; Paul et al., 2020). Consistent with patterns observed in *Ascomycota*, these endobacteria retain genome sizes comparable to those of their free-living counterparts (Glaeser et al., 2016; Nag and Seal, 2024). Endomicrobial diversity also remains high; however, a clearer correlation begins to emerge between the presence of specific taxa and distinct adaptive advantages, particularly under selective pressure.

The next level of integration is observed in *Glomeromycota*, where endosymbiotic associations exhibit a long evolutionary history and a higher degree of functional coordination. In systems such as *G. margarita*, vertical transmission and coordinated regulation involving lipid metabolism and defense responses among the fungus, the bacteria, and even the plant host have been documented (Bianciotto et al., 2004; Venice et al., 2017; Venice et al., 2021). Moreover, the coexistence of multiple endosymbionts, such as *Ca*Gg and *Ca*Mg, together with progressive genome reduction within a single host, represents one of the earliest indications of a structured fungal microbiome, displaying adaptive capacity and genetic plasticity that may facilitate transitions toward more integrated states (Desirò et al., 2014; Mugambi et al., 2026).

In *Mucoromycota*, these interactions reach a more advanced level of co-dependence. The model involving *R. microsporus*, *M. rhizoxinica*, and *M. endofungorum* demonstrates direct functional integration encompassing essential processes such as reproductive development, including sporulation (Partida-Martínez et al., 2007c; Mondo et al., 2017), biosynthetic pathways associated with toxin production (Partida-Martinez and Hertweck, 2005), and specialized colonization mechanisms (Moebius et al., 2014; Lastovetsky et al., 2020). In this system, endobacteria can still be cultured independently; nevertheless, they exhibit genome reduction, suggesting an intermediate stage in the progression toward increased dependency (Abbot et al., 2025).

In *Mortierellomycota*, host reliance becomes further intensified. Endosymbionts such as *M. cysteinexigens*, which have not been successfully isolated from their host, lack essential metabolic pathways, including those involved in cysteine biosynthesis and cofactor production, reflecting an extensive interdependence with the fungal partner (Uehling et al., 2017; Sharmin et al., 2018). Although removal of the endosymbiont is not lethal to the fungus, it significantly alters host metabolism, reflecting an advanced level of functional integration (Li et al., 2017). Furthermore, the presence of additional endosymbionts with extremely reduced genomes, such as *MRE* populations, further supports the notion of progressive genetic simplification associated with intracellular lifestyles (Longley et al., 2023).

Finally, in *Zoopagomycota*, highly specialized associations are observed, which may represent more functionally ancient states. Although studies remain limited, available evidence points to the presence of specific, non-cultivable bacterial groups, with potential gene transfer events to the fungal host. In insect-associated systems, such as those involving aphids, these interactions form part of complex symbiotic networks spanning multiple trophic levels, in which specialized structures for housing microbiota have even evolved (Chen et al., 2016). This degree of integration suggests advanced states of stabilization, where initial diversity has been reduced to highly specific and functionally defined associations.

Importantly, some systems display traits that do not fit neatly within a progressive trajectory of integration. For example, genome reduction is not observed in *N. punctiforme* associated with *G. pyriformis*, despite the coexistence of highly reduced *MRE* populations within the same host system (Sorwar et al., 2024). Likewise, cultivable endosymbionts reported in non-model hosts such as *R. oryzae* and *Mucor* spp. remain functionally poorly characterized, limiting current interpretation of their degree of integration (Birol and Gunyar, 2021; Okrasińska et al., 2021).

Experimental models of artificial endosymbiosis support the model of progressive endosymbiosis. In engineered yeast systems, intracellular persistence, metabolic interdependence, and early stages of genomic adaptation have been shown to emerge over relatively short evolutionary timescales under defined selective pressures. The involvement of host cellular machinery suggests that the stabilization of these associations requires the progressive co-option of host cellular processes (Mehta et al., 2018; Gao et al., 2024). Moreover, not all bacterial lineages exhibit the same capacity to establish stable intracellular relationships, indicating the existence of phylogenetic preadaptations that shape the trajectory toward integration (Uribe-Alvarez et al., 2019). Complementary approaches, including chemically induced colonization and synthetic systems such as fungus-alga associations, further underscore the plasticity of these interactions. Collectively, these findings reinforce the concept that endosymbiosis is not a discrete event, but rather a dynamic and experimentally reproducible evolutionary *continuum*.

Taken together, these patterns suggest an evolutionary trajectory in which endosymbiosis begins as an open and dynamic process, characterized by the frequent incorporation of microorganisms, and progressively advances toward states of specialization, genome reduction, and metabolic dependence. At more advanced stages, this process may involve gene transfer to the host genome and the loss of endosymbiont autonomy, that may contribute specialized functions resembling early stages of proto-organelles, as described in other microbial eukariotic hosts (Marks et al., 2025).

## 6 Conclusion

Integrated evidence across fungal lineages indicates that endosymbiosis constitutes an evolutionary *continuum*, progressing from dynamic and highly diverse associations to highly specialized, stable, and functionally integrated interactions. This gradient underpins the theory of progressive endosymbiosis, in which the initial incorporation of microorganisms can evolve toward states of metabolic dependence, genome reduction, and, ultimately, genetic integration with the host.

Fungal systems emerge as valuable models for studying intermediate stages of intracellular integration, providing evidence that the transition from flexible symbiotic associations toward increasingly specialized endosymbiotic states may represent a gradual and potentially recurrent evolutionary process. In this regard, advances in artificial endosymbiosis further support the feasibility of inducing and studying these trajectories under controlled conditions. Collectively, these findings not only redefine the role of endosymbiosis in eukaryotic evolution, but also position fungi as key platforms for the design of intracellular systems with high-impact biotechnological applications.
